# Characterization of Partial Ligation-Induced Carotid Atherosclerosis Model Using Dual-Modality Molecular Imaging in ApoE Knock-out Mice

**DOI:** 10.1371/journal.pone.0073451

**Published:** 2013-09-12

**Authors:** Ik Jae Shin, Soo-Min Shon, Dawid Schellingerhout, Jin-Yong Park, Jeong-Yeon Kim, Su Kyoung Lee, Dong Kun Lee, Ho Won Lee, Byeong-Cheol Ahn, Kwangmeyung Kim, Ick Chan Kwon, Dong-Eog Kim

**Affiliations:** 1 Molecular Imaging and Neurovascular Research (MINER) Laboratory, Dongguk University College of Medicine, Goyang, Korea; 2 Departments of Radiology and Cancer Systems Imaging, University of Texas M. D. Anderson Cancer Center, Houston, Texas, United States of America; 3 Laboratory of Genome to Drug Medicine, Joint Center for Biosciences, Incheon, Korea; 4 Department of Nuclear Medicine, School of Medicine, Kyungpook National University, Daegu, Korea; 5 Biomedical Research Center, Korea Institute of Science and Technology, Seoul, Korea; Brigham and Women's Hospital, Harvard Medical School, United States of America

## Abstract

**Background:**

Recently, partial ligation of the common carotid artery (CCA) was reported to induce carotid atheromata rapidly in apolipoprotein-E knockout (ApoE^-/-^) mice. We investigated this new atherosclerosis model by using combined matrix-metalloproteinase (MMP) near-infrared fluorescent (NIRF) imaging and macrophage-tracking luciferase imaging.

**Methodology and Principal Findings:**

Partial ligation of the left CCA was performed in 10-week-old ApoE^-/-^ mice on a high fat diet (n=33); the internal and external carotid arteries and occipital artery were ligated, while the superior thyroid artery was left intact. Two thirds of the animals were treated with either LiCl or atorvastatin. At 1-week, Raw264.7 macrophages modified to express the enhanced firefly-luciferase reporter gene (10^7^ Raw-luc cells) were injected intravenously. At 2-week, NIRF molecular imaging visualized strong MMP-2/9 activity in the ligated area of the left CCA as well as in the aortic arch. Left-to-right ratios of the NIRF signal intensities in the CCA had a decreasing gradient from the highest value in the upper-most ligated area to the lowest value in the lower-most region adjacent to the aortic arch. Luciferase imaging showed that most Raw-luc macrophages were recruited to the ligated area of the CCA rather than to the aortic arch, despite similarly strong MMP-2/9-related NIRF signal intensities in both areas. In addition, LiCl or atorvastatin could reduce MMP-2/9 activity in the aortic arch but not in the ligated area of the CCA.

**Conclusions/Significance:**

This is the first molecular imaging study to characterize the partial ligation-induced carotid atherosclerosis model. Molecularly divergent types of atherosclerosis were identified: conventional lipogenic atherosclerosis in the aorta vs. flow-related mechanical atherosclerosis in the partially ligated left system.

## Introduction

Nam et al recently reported that partial ligation of the left common carotid artery (CCA) induced carotid atheromata rapidly over 2 to 4 weeks in apolipoprotein E knockout (ApoE^-/-^) mice fed on a western diet by causing turbulent flow and low and oscillatory shear stress in the artery [1]. By blocking the normal flow of blood through the carotid system at all outflow points except for the superior thyroidal artery, significant stress is placed on the vascular endothelium leading to the activation of pro-atherogenic and anti-atherogenic genes as well as mechanosensitive genes [2]. Ligation also rapidly stimulated the recruitment of leukocytes, such as monocytes / macrophages and T cells, to the carotid arterial wall within 7 days [3]. However, further characterization of the new atherosclerosis model is still required: monitoring response to anti-atherosclerotic interventions as well as analyzing the extent and degree of atherosclerotic lesion development along the carotid artery below the ligated part.

Proteases such as matrix metalloproteinases (MMPs) secreted by macrophages could render atherosclerotic plaques unstable and prone to rupture and thereby cause sudden thromboembolic occlusion [4]. The matrix-degrading activity of MMPs is also essential for pathological arterial remodeling in atherosclerosis and restenosis [5,6]. In C57BL/6 mice, carotid artery flow cessation resulted in an early significant upregulation of MMP-9 expression and expansive remodeling [7,8]. We previously showed that near-infrared fluorescence (NIRF) imaging using a protease-activatable probe enabled quantitative mapping of in vivo cathepsin-B or MMP-2/9 protease activity in atheromata, reflecting the inflammatory component of atherosclerotic pathology in mice [9] and human atheromata [10]. We also showed that the protease imaging could demonstrate plaque-stabilizing effects of anti-atherosclerotic drugs such as atorvastatin [11] and treadmill exercise training [8] in mice. There is a need for the molecular imaging technology to be applied to the characterization of atherosclerosis animal models.

In this study to utilize a combined bioluminescent and NIRF molecular imaging technique, we characterized the new partial ligation-induced carotid atherosclerosis model by 1) imaging recruitment of luciferase-transfected macrophages to atheromata in vivo / ex vivo and MMP-2/9 activity within the atheromata ex vivo and 2) estimating anti-atherosclerotic effects of atorvastatin and LiCl.

## Materials and Methods

### Ethics Statement

This study was approved by the Animal Care and Use Committee of Dongguk University Ilsan Hospital. All experiments were performed in accordance with the National Institutes of Health guidelines for the care and use of laboratory animals. Surgical sites were cleaned with 70% alcohol, followed by povidone iodine. In order to prevent intra-operative hypothermia and post-operative shivering, a thermistor-controlled heating blanket and temperature-supported cage were used, respectively. Animals were euthanized after inhalation anesthesia and blood collection (~ 2ml).

### Synthesis of MMP-2/9 activatable molecular imaging probe

A polymeric nanoparticle-based MMP-2/9 activatable probe was synthesized and characterized as described previously [10,11]. An MMP-2/9 cleavable NIRF dye-peptide-quencher substrate, Cy5.5-Glu-Leu-Pro-Gly-Arg-Gly-Lys(BHQ-3)-Gly-Gly-COOH, was conjugated to glycol chitosan nanoparticles. The resulting particles were spherical and approximately 250nm in diameter. The imaging probes were well dispersed in the reaction buffer (100 mM Tris, 5 mM calcium chloride, 200 mM NaCl, 0.1% Brij, pH 7.5), and quenching of NIRF signal was confirmed using a Cy5.5 NIRF filter set and a small animal imager (Kodak Image Station 4000MM, Kodak, Rochester, NY).

### Preparation and characterization of Raw264.7 macrophages expressing the enhanced firefly luciferase (effluc) gene

Murine macrophage Raw264.7 cells (American Type Culture Collection, Manassas, VA) were kindly provided by Dr. C-W Kim (Seoul National University, Seoul, Korea) and were stably transfected to express the effluc gene by transducing with the retrovirus to express both effluc and Thy1.1 genes [12]. The resulting stable cell line expressing the effluc gene was characterized and is referred to as the Raw-luc cell line. For the detailed information, please see the Supplementary Methods (File S1).

### Animals and experimental procedures

Ten-week-old ApoE^-/-^ mice (26~30 g, n=33) were purchased (Jan-SLC, Shizuoka, Japan) and were maintained in a controlled environment of 20°C and 40~50% humidity, with 12 h of light per 24 h period. The western diet and water were available *ad libitum*. Partial ligation of the left CCA was performed as previously described [1]. Briefly, anesthesia was induced by 2% isoflurane inhalation. The neck was epilated and then disinfected with 70% ethanol solution. A ventral midline incision (4~5 mm) was made in the neck. The left CCA and the right CCA were exposed by blunt dissection. Three out of the four caudal branches of the left CCA (the external carotid, internal carotid, and occipital artery) were ligated with a 6.0 silk suture, while the superior thyroid artery was left intact. The right CCA was not ligated and served as an internal control. Two thirds of the animals were treated with either LiCl (5 mM mixed with drinking water; n=11) or atorvastatin (mixed with diet, 0.01% w/w; n=11). At one week after the carotid ligation, Raw-luc macrophages (1x10^7^) were injected intravenously. At two weeks, 4 µM (200 µL) MMP-2/9 activatable NIRF probe was intravenously injected. Four hours later, the neck was opened to expose the CCAs, and D-luciferin (150 mg/kg; PerkinElmer, Santa Clara, CA) was injected intraperitoneally and in vivo bioluminescent imaging (600-seconds acquisition) was performed using the IVIS-200 small animal imaging system (PerkinElmer, Waltham, MA). Then, the animals were euthanized, and the CCAs and aorta were carefully excised *en bloc*. The tissue was washed with PBS three times, and combined bioluminescent imaging (300-seconds acquisition) and MMP-2/9 NIRF imaging (excitation/emission, 675/690 nm; 1-second acquisition) were performed ex vivo. After this final imaging session, the CCAs and aortic root were snap-frozen in OCT compound, and the remaining aortic tissue was embedded in paraffin. Fresh frozen tissues were stored at -80°C until further use.

### Image quantification

**Figure 1:**
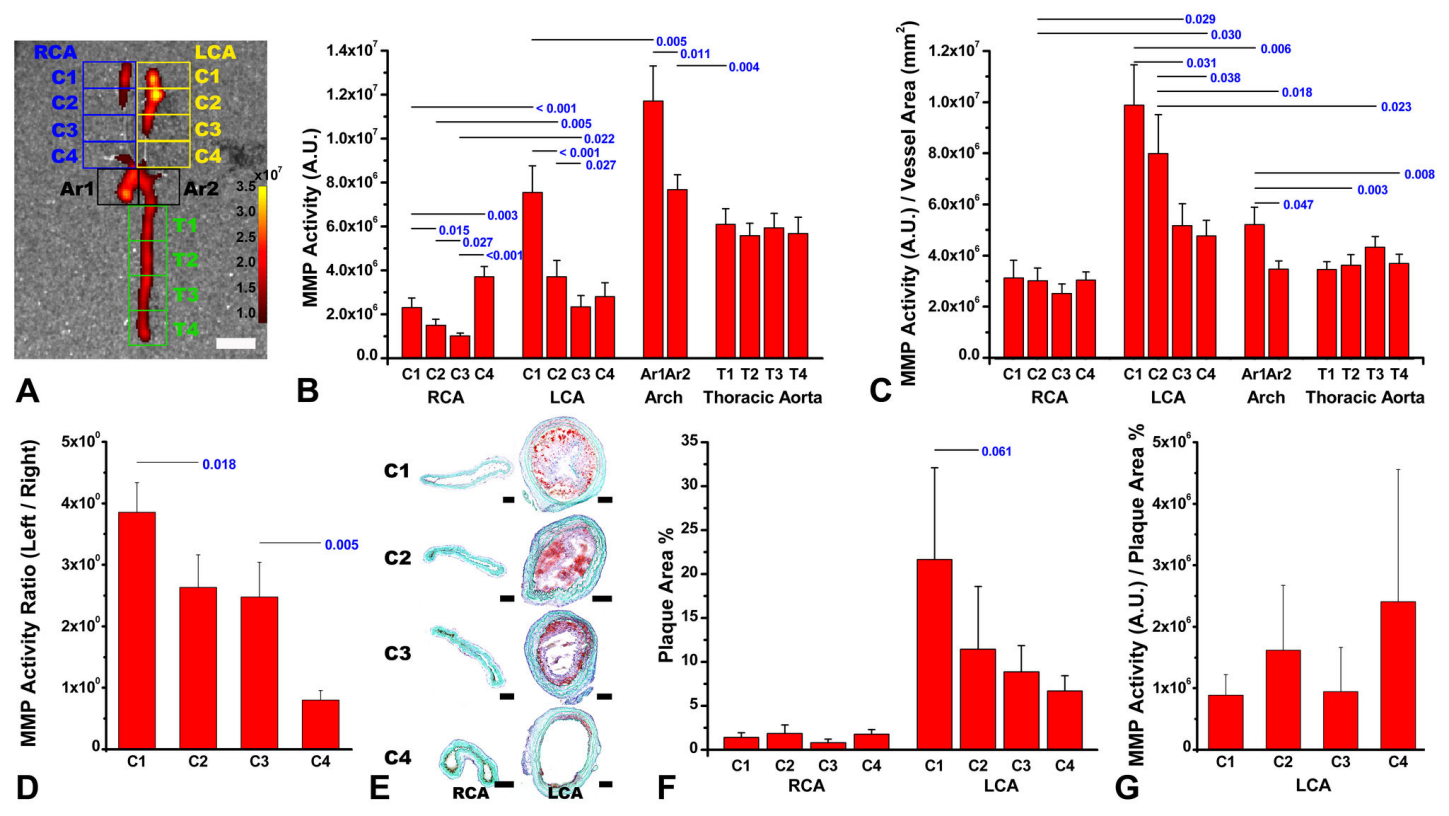
Strong matrix metalloproteinase (MMP)-2/9-related near-infrared fluorescent (NIRF) signal in and around the ligated area of the left common carotid artery (LCA) of ApoE^-/-^ mice fed on a western diet. Two weeks after partial ligation of the LCA, there is a gradient of MMP-related fluorescence (mean intensity / tissue area in a rectangular region of interest) that is maximal close to the ligation site / bifurcation point (A, LCA C1 or C2), and progressively decreased as one move inferiorly closer to the arch both on the right and on the left, but the maximum is much higher on the ligated left side (A and B). The arch itself shows the highest activities measured, particularly proximally, close to the aortic root (B, Ar1 vs. Ar2). However, the MMP activity per vessel area in each square region of interest is highest in the LCA ligation site (C, LCA C1 and C2). There is a decreasing gradient in the left-to-right ratios of the NIRF signal intensities (D) and oil red O staining-positive atherosclerotic lesion size (E and F) in the four carotid segments. MMP activity / plaque size ratios in the left CCA do not show significant regional differences (G). Pseudo-color overlaid NIRF signal in the aorta and arteries with intensity expressed as arbitrary unit (A). RCA denotes the right common carotid artery. Statistically significant or marginally significant p values from paired-t tests are provided. White scale-bar, 1 mm; black scale-bars, 100 µm.

**Figure 2:**
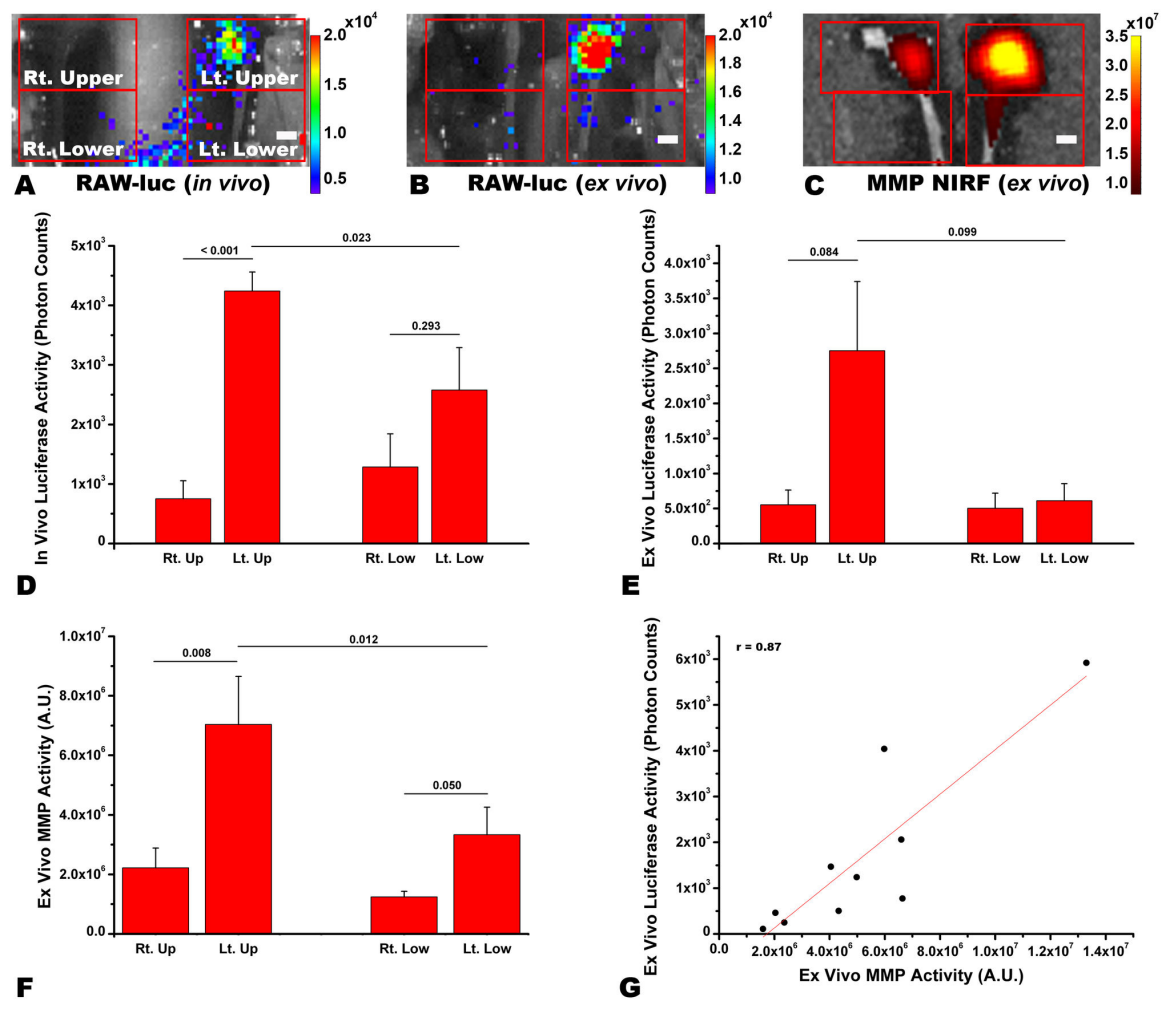
Recruitment of intravenously-injected Raw-luc macrophages to the ligated area of the left common carotid artery (CCA) with strong matrix metalloproteinase (MMP)-2/9 activity. Two weeks after partial ligation of the left CCA and one week after intravenous injection of Raw-luc cells in the representative ApoE^-/-^ mouse, in vivo (A) and ex vivo (B) bioluminescence imaging with pseudo-color overlay (photon counts / second) shows a clustering of strong luciferase signal in and around the ligated area of the left CCA, where strong MMP-2/9-related signal is observed on ex vivo near-infrared fluorescent (NIRF) imaging with pseudo-color overlay (C; signal intensity, arbitrary unit). Quantitative data (D to F) for the four quadrants in A to C corroborate the above findings. In the left CCA, there is a linear correlation between the photon counts (per second / quadrant) on the ex vivo luciferase imaging and NIRF signal intensities on the ex vivo MMP imaging (r = 0.87, p = 0.001, Pearson correlation). Statistically significant p values from paired-t tests are provided. Scale-bar, 1 mm.

**Figure 3:**
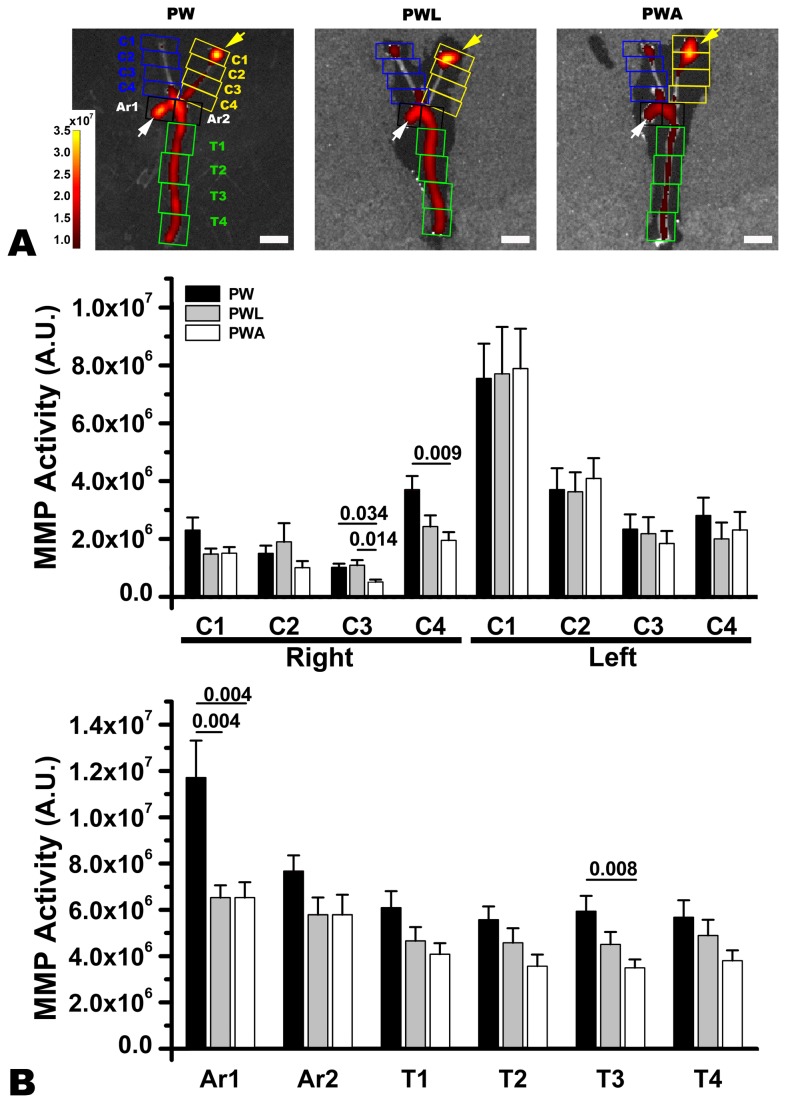
LiCl or atorvastatin treatment to attenuate matrix metalloproteinase (MMP)-2/9-related NIRF signal intensities in the aortic arch, but not in the ligated area of the common carotid artery (CCA). Two weeks after partial ligation of the left CCA, lower MMP-2/9-related NIRF signal intensities are observed in the proximal part of the aortic arch of the representative ApoE^-/-^ mouse fed on a western diet with LiCl (PWL) or atorvastatin (PWA) than in that of the animal without treatment (PW) (white arrows); however, no inter-group differences are observed in the ligated area of the CCA (yellow arrows). Pseudo-color overlay with numbers (arbitrary unit) represents signal intensities (arbitrary unit). Quantitative data (B) corroborate the above findings. MMP activity was also attenuated in the proximal right CCA near the aortic arch (C3 and C4) and descending aorta (T3) of the western diet-fed mice treated with atorvastatin. Statistically significant p values from paired-t tests are provided. Scale-bar, 5 mm.

Quantification of NIRF signal (mean intensity; arbitrary unit, A.U.) and bioluminescence signal (photon counts / second) was performed as previously reported [9-11,13,14] in the CCAs (2 or 4 equi-length segments) and aorta (entire aorta and aortic subdivisions) (Figures 1 to 3) using Living Image software (PerkinElmer, Waltham, MA).

### Histology and quantification

Quantification of atherosclerotic lesion size (n = 5 animals / group) was performed as previously published [6,8] using 10 µm-thick transverse CCA sections (n = 16/4 equi-length segments / animal) stained with oil red O. Values are reported as the percentage of the carotid tissue covered by atherosclerotic lesions divided by the total area of the carotid tissue. Quantification of vascular remodeling was performed by measuring the greatest intima / media thickness measurement as well as the media / lumen area, using equidistant carotid (n = 4 / animal, transverse) and aortic (n = 4 / animal, longitudinal) sections (10µm thick) that were stained with oil red O and light green. Adobe Photoshop CS-3-Extended (Adobe Systems, San Jose, CA) was used to segment and measure lesion size. Immmunohistochemistry for Mac-3 or MMP-9 was performed using the avidin-biotin-peroxidase method as previously reported [9,11]. Immuno-positive areas were quantified (n = 5 animals / group) by using equidistant carotid (n = 4 / animal, transverse) and aortic (n = 4 / animal, longitudinal) sections (10 µm thick). The extent of brown-colored immuno-positive areas was quantified with the color range function and measured using the histogram function of Adobe Photoshop.

### Data analysis

Data are presented as mean ± standard error. The SPSS software package (SPSS 18.0, Chicago, IL) was used to perform paired t-tests, Wilcoxn signed-rank tests, and Mann-Whitney tests.

## Results

### Strong MMP-2/9-related NIRF signal in and around the ligated area of the CCA of ApoE^-/-^ mice fed on a western diet

Two weeks after partial ligation of the left CCA, NIRF molecular imaging detected strong MMP-2/9-related protease activity in and around the ligated area of the left CCA and aortic arch (Figure 1A). Relatively weak MMP-2/9-related NIRF signal was observed in the lower segments below the ligated area of the left carotid artery. In the non-ligated right carotid artery, weak NIRF signal was observed in the most proximal region adjacent to the aortic arch or in the carotid bifurcation area. In the other areas of the right CCA, NIRF signal was rarely observed.

Quantitative data (Figure 1B, n = 11) corroborated the above findings. The MMP-2/9-related NIRF signal intensity in the ligated area of the left CCA was similar to that noted in the distal half of the aortic arch and slightly lower than that of the proximal half of the aortic arch. The MMP activity per vessel area was significantly higher in the ligated area of the left CCA than in the aortic arch (Figure 1C). Left-to-right ratios of the NIRF signal intensities in the four carotid segments appeared to have a decreasing gradient: highest in the upper-most ligated area, lower in the below two segments, and lowest in the lower-most region adjacent to the aortic arch (Figure 1D).

Oil red O staining of a representative animal (Figure 1E) showed that atherosclerotic lesion size was bigger and lumen size was smaller in the ligated area than in the more proximal areas of the left carotid artery. Quantitative studies (Figure 1F and G) corroborated this: the closer to the ligated area, the higher the intimal thickness and luminal narrowing. In the right carotid artery, oil red O staining-positive lesions were scarce (Figure 1E and F).

There were no regional differences in the MMP activity / plaque size ratios in the ligated left CCA (Figure S1 in File S2).

### Combined bioluminescence / fluorescence imaging to demonstrate the recruitment of intravenously-injected Raw-luc macrophages to the ligated area of the CCA with strong MMP-2/9-related NIRF signal

Two weeks after partial ligation of the left CCA and one week after intravenous injection of Raw-luc cells (please see Figures S2 and S3 in File S2 for characterization data), in vivo bioluminescence imaging demonstrated a clustering of strong luciferase signal in and around the ligated area of the left CCA (Figure 2A). Similar findings were observed in the following ex vivo bioluminescence imaging (Figure 2B). Ex vivo NIRF imaging demonstrated that the area with luciferase signal also had strong MMP-2/9-related signal (Figure 2C). In the aortic arch, luciferase signal was rarely observed (Figure S4 in File S2).

Quantitative analyses (n=5) of the in vivo (Figure 2D) or ex vivo (Figure 2E) luciferase imaging data showed that photon counts were about four- or five-fold higher in the upper half of the left CCA, which contained the ligated area, than in either the upper or lower half of the non-ligated right CCA. In the lower half of the left CCA, photon counts appeared to be higher in the corresponding lower half of the right CCA, which however did not reach a statistical significance. Similarly, in the quantitation of the combined ex vivo MMP imaging data (Figure 2F), NIRF signal intensities were highest in the upper half of the left CCA, followed by the lower half of the left CCA. In the left CCA, with the upper and lower parts combined, there was a linear correlation between the photon counts on the ex vivo luciferase imaging and NIRF signal intensities on the ex vivo MMP imaging, (Figure 2G; p = 0.001, r = 0.87). In the right CCA, there was no significant linear correlation between the two variables (p>0.05, data not shown).

### **MMP-2/9-related NIRF signal intensities being significantly attenuated with LiCl or atorvastatin treatment for two weeks in the aortic arch, but not in the ligated area of the CCA**

Two weeks after partial ligation of the left CCA in the ApoE^-/-^ mice fed on a western diet and treated with LiCl (n = 11) or atorvastatin (n = 11) vs. tap water control (n = 11), MMP-2/9-related NIRF signal intensities did not show inter-group differences in and around the ligated area of the left CCA (yellow arrows in Figure 3A and Figure 3B). In the proximal half of the aortic arch however, NIRF signal intensities were lower (white arrows in Figure 3A and Figure 3B) in the western diet-fed mice treated with LiCl or atorvastatin than in the controls on a western diet and tap water. In the proximal right CCA near the aortic arch (Figure 3B), and descending aorta (Figure 3B), NIRF signal intensities were attenuated by statin, but not by LiCl treatment.

**Figure 4:**
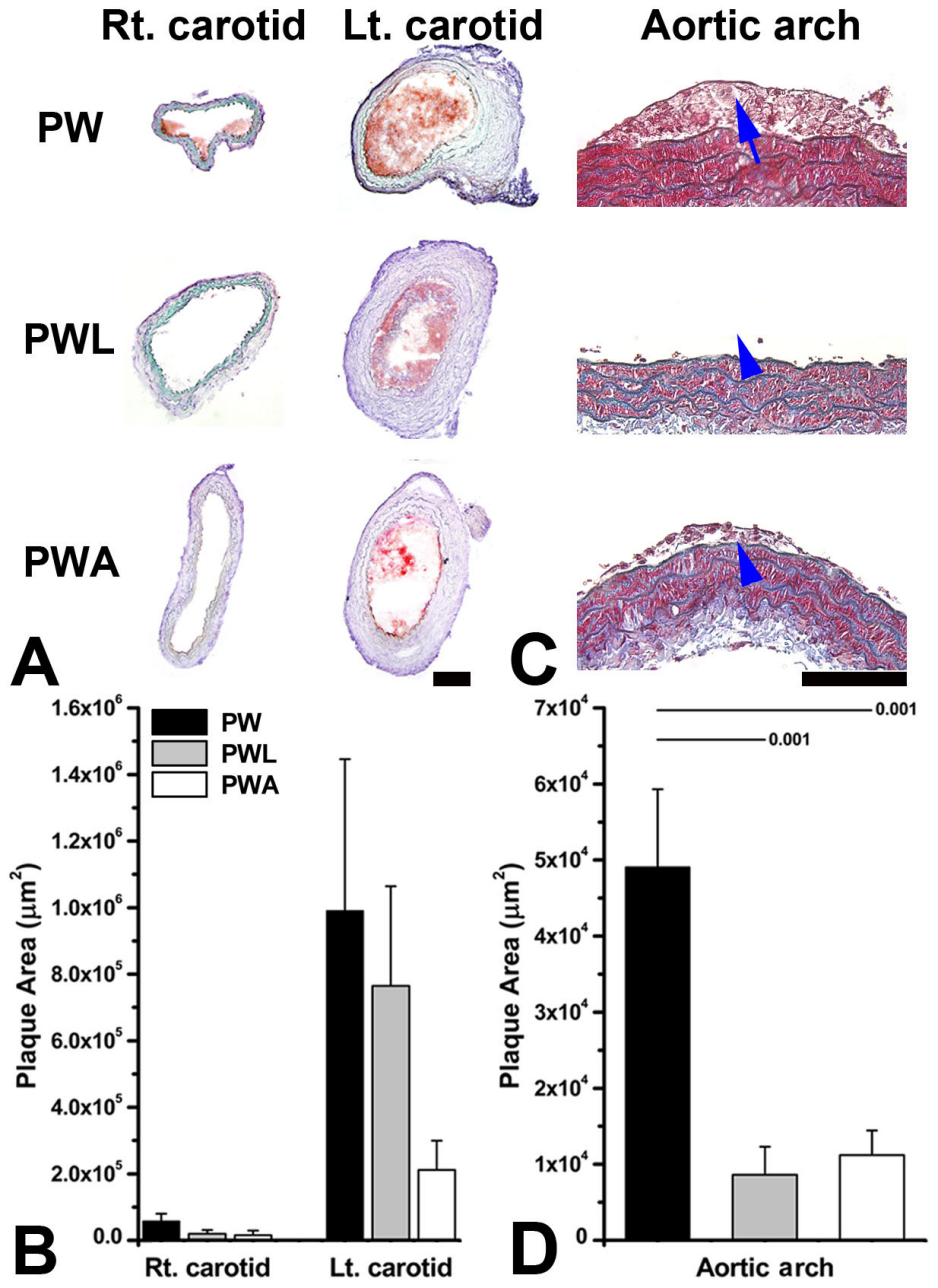
LiCl or atorvastatin to decrease plaque size significantly in the aortic arch, but not in the ligated area of the left common carotid artery (CCA). Two weeks after partial ligation of the left CCA, carotid plaque size (oil red O-stained red area) was non-significantly smaller in the representative ApoE^-/-^ mouse fed on a western diet with LiCl (PWL) or atorvastatin (PWA) treatment vs. the animal without treatment (PW) (A). This is corroborated by the quantitative data (B). In the aortic arch (C, Masson’s trichrome staining), plaque size is significantly smaller in the treated animals (blue arrow-heads) than in the control (blue arrow). Quantitative data (D) again corroborate the findings. Statistically significant p values from Mann-Whitney tests are provided. Scale-bars, 100 µm.

In the ligated area of the left CCA, plaque size was non-significantly smaller in the western diet-fed mice treated with atorvastatin or LiCl than in the control animals on a western diet and tap water (Figure 4A and B). In the proximal aortic arch, plaque size was significantly smaller in the western diet-fed mice treated with LiCl or atorvastatin than in the controls on a western diet and tap water (arrow-heads in Figure 4C and Figure 4D).

**Figure 5:**
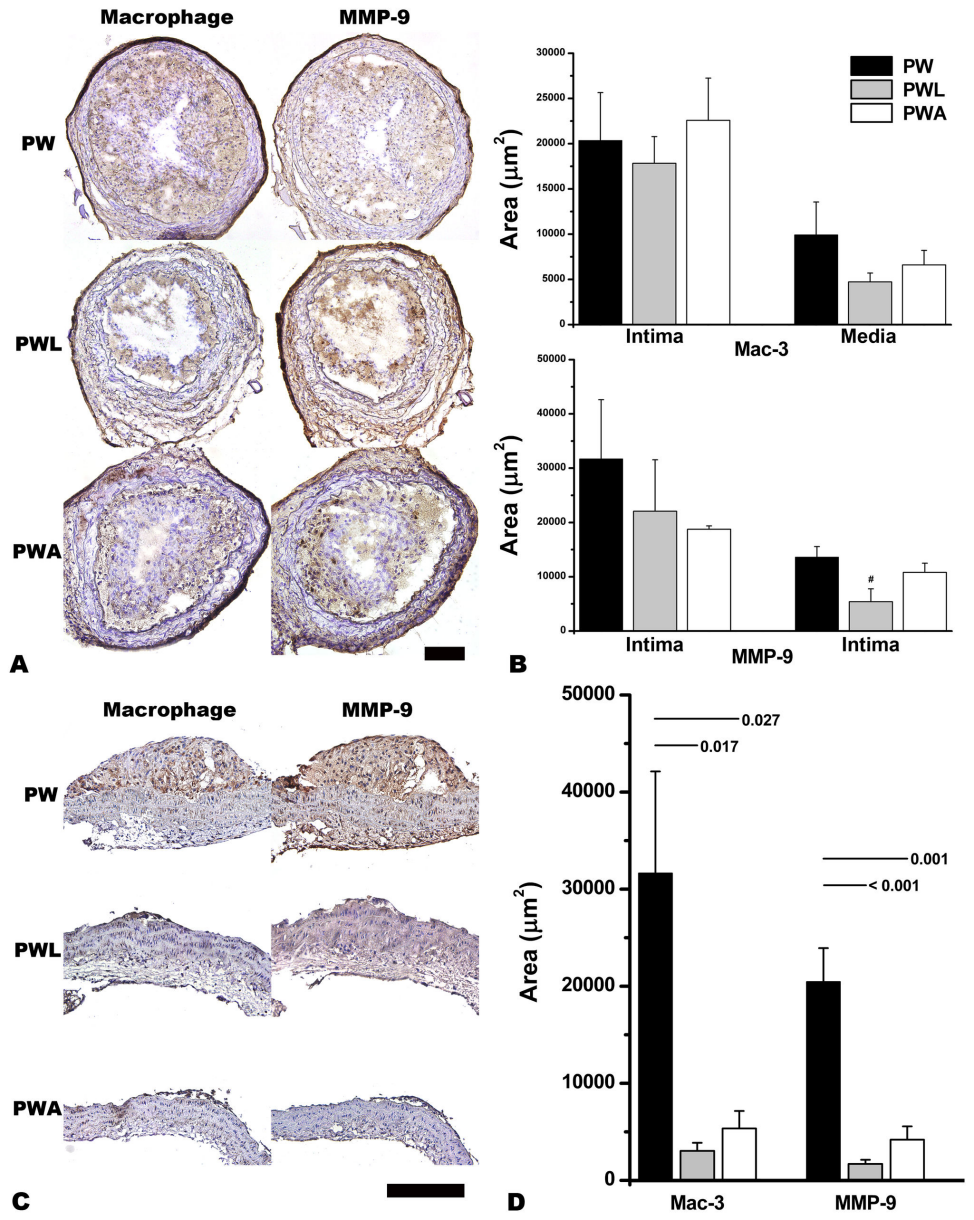
LiCl (PWL) or atorvastatin(PWA)-induced attenuation of plaque size and the extent of macrophage (Mac-3) or matrix metalloproteinase-9 (MMP-9) immunoreactivity in the aortic arch (C and D) but not in the left common carotid artery (A and B). PW denotes non-treated western-diet fed ApoE^-/-^ mice with partial ligation-induced carotid atherosclerosis. Statistically significant p values from Mann-Whitney tests between groups are provided. MMP-9 immunoreactivity in the intima tends to be less extensive in the PWL group than in the other groups (B, ^#^ p = 0.083). Scale-bars, 100 µm.

The extent of Mac-3 or MMP-9 immunoreactivity did not show significant inter-group differences in the ligated area of the CCA (Figure 5A and B). In the proximal half of the aortic arch however, plaque size and Mac-3 or MMP-9 immunoreactivity were decreased in the western diet-fed mice treated with LiCl or atorvastatin compared with the mice on a western diet and tap water (Figure 5C and D).

## Discussion

This is the first study to characterize the partial ligation-induced carotid atherosclerosis model using a combined NIRF and bioluminescent molecular imaging technique. We demonstrate the distribution of in vivo MMP activity along the length of the CCA and aorta as well as recruitment of macrophages to atherosclerotic lesions. Our observations show that conventional lipogenic atherosclerosis and high-flow mechanical atherosclerosis acts similarly and differently in interesting ways. Specifically we found: a) MMP-related NIRF signal to be present in both types of atherosclerosis, b) Macrophage chemotaxis was far higher in mechanical atherosclerosis than in lipogenic atherosclerosis, and c) treatment paradigms successful for lipogenic atherosclerosis (lithium and atorvastatin) failed to affect the vascular modeling observed in mechanical atherosclerosis.

Two weeks after the CCA ligation, strong MMP-2/9-related NIRF signal was observed in the ligated area, where strong luciferase signal from intravenously injected Raw-luc macrophages was also observed. Below the ligated area down to the CCA origin near the arch, relatively weak MMP signal was observed. However, the NIRF signal was relatively strong when compared with the corresponding part of the non-ligated right CCA, except for the CCA origin. Left-to-right ratios of the NIRF signal intensities appeared to have a decreasing gradient from the ligated area to the origin of the CCA, reflecting the regional pattern of the carotid ligation-induced vascular remodeling: the closer to the ligated area, the higher the intimal thickness and luminal narrowing.

MMP activity was increased not only in the ligated area but also in the lower proximal part of the CCA, which can be partly explained by the results of the computational fluid dynamics study by Nam et al.: partial ligation causes low and oscillatory shear stresses to be evenly distributed through the length of the CCA, both proximal and distal to the ligation site [1]. In our study however, the ligated area was observed to have the highest MMP activity and the most severe vascular remodeling, with a decreasing gradient down to the origin of the CCA. Thus, in addition to changes in shear stress levels and direction by carotid ligation-induced arterial flow disturbance [15-17], other contributing factors should be considered, such as infiltration of macrophages that are regarded as key cellular protagonists of atherosclerosis [18-20].

In line with the non-uniform development of CCA atherosclerosis in this model, the recruitment of Raw-luc macrophage cells to the left CCA was higher in the upper half than in the lower half. Total MMP activities were similarly high in the ligated area of the CCA and in the aortic arch. However, compared with the accelerated [21-23] form of CCA atheromata, in the spontaneous form of aortic atheromata bioluminescent signal from Raw-luc macrophages was less frequently observed, suggesting that the model presents two types of atherosclerosis pathology [24].

LiCl or atorvastatin could significantly reduce in vivo MMP-2/9 activity in the aortic arch but not in the ligated area of the CCA, reflecting that the model has two types of vascular pathology: ApoE deletion and high fat diet-induced primary atherosclerosis vs. flow disturbance-mediated accelerated vascular remodeling. Recently, in 10-week-old ApoE^-/-^ mice on a high fat diet (20% fat and 0.5% cholesterol), supplementation with LiCl for 6 or 14 weeks was reported to reduce atherosclerotic lesion formation in the aorta [25]. In the present study, when treated with LiCl for only 2 weeks, plaque size was smaller and in vivo MMP activity was lower in the proximal aorta of 10-week-old ApoE^-/-^ mice fed on a high fat diet (21% fat and 1.5% cholesterol) compared with the animals without treatment. The anti-atherosclerotic effect of LiCl was comparable to that of atorvastatin.

Unexpectedly, in the carotid arteries neither of plaque size or MMP activity was significantly affected by LiCl or atorvastatin. Notably, Ali et al. reported that after exposure to oscillatory flow endothelial cells showed significant attenuation of the atorvastatin-mediated expression of heme oxygenase-1 [26], which may have a protective role during atherogenesis by inhibiting vascular smooth muscle cell proliferation and by exerting anti-inflammatory, vasodilatory, and antioxidant effects [27]. Thus, they suggested that anti-inflammatory pleiotropy of statins may be suboptimal at atherosusceptible sites with disturbed flow [26]. In our study, the divergent therapeutic response of CCA vs. aortic atheromata suggests again that the two lesions are pathophysiologically different. Further comparative studies are required to clarify this.

In the previous study to estimate potential clinical efficacy of the MMP-2/9 activatable NIRF probe, we reported observational evidence that plaque vulnerability could be assessed by means of NIRF molecular optical imaging of carotid endarterectomy tissues from elderly patients, which complements and adds to traditional anatomic information such as angiography or ultrasonography [10]. Recently, using the same molecular imaging probe, we demonstrated that a 10-week exercise training program in the 17-week-old ApoE^-/-^ mice with preexisting atheromata reduced plaque-destabilizing MMP activity, even if animals remained on a high fat diet and plaque-growth was not attenuated [11]. This kind of informative divergence between morphological data and molecular imaging data was not observed in the present study; in vivo MMP activity was proportional to the corresponding plaque size. In the rapidly formed carotid atheromata of relatively young mice, anatomic phenotypes such as plaque size and underlying molecular events such as MMP activity [28] might be too closely interlinked to be differentially affected by short-term anti-atherosclerotic therapy.

Our study is limited by the inevitable divergences between mouse and human pathophysiology, but point to the very important differences also observed in humans between flow-induced mechanical vascular disease and lipogenic atherosclerosis [7]. It hints at the convergence in these two disparate types of atherosclerosis in diseases such as carotid stenosis, where lipogenic atherosclerosis merges with mechanical disease to accelerate the final phases of vascular disease leading up to stroke. Although there is still much more work to be done, the data we demonstrated may justify further research into this area.

In conclusion, we used dual-modality molecular imaging with an activatable MMP-2/9 probe and Raw-luc macrophages to characterize a new mouse model with two different types of atherosclerotic vascular disease: conventional lipogenic atherosclerosis and shear related mechanical atherosclerosis. We show that these two types of vascular disease are different in terms of their molecular characterization, with both types showing MMP related remodeling, but with far greater macrophage chemotaxis in mechanical atherosclerosis. This has important therapeutic implications, and indeed we show that treatments such as lithium or atorvastatin are effective for conventional lipogenic atherosclerosis, but not for mechanical atherosclerosis. We believe that this animal model and the molecular imaging techniques used to study it will yield significant insights in the molecular dissection of atherosclerotic disease.

## Supporting Information

File S1 Supplementary methods. (DOC)

File S2 File includes Figures S1-S4. (PDF)
