# Supplementary material for: Characterization of Partial Ligation-Induced Carotid Atherosclerosis Model Using Dual-Modality Molecular Imaging in ApoE Knock-out Mice
Source: PLoS One. 2013 Sep 12;8(9):e73451. doi: 10.1371/journal.pone.0073451 (PMC3772018; doi:10.1371/journal.pone.0073451)
Supplement: File S1 — (DOC) [file pone.0073451.s001.doc]

**Supplementary Methods**

Preparation of Raw264.7 macrophages expressing the enhanced firefly luciferase (effluc) gene

Murine macrophage Raw264.7 cells were stably transfected to express the effluc gene by transducing with the retrovirus to express both effluc and Thy1.1 genes. pRetro/LTR-effluc-IRES-Thy1.1 vector was a kind gift from Dr. Patrick Hwu (M.D. Anderson cancer center, TX), and the retrovirus co-expressing effluc and Thy1.1 genes was produced by using a reported protocol [1]. Briefly, 2 × 10^6^ phoenix cells were plated cells on 100 cm tissue culture plates and transfected with pMSCV-ffFLuc-pIRES-Thy1.1 using a Calcium Phosphate Transfection Kit (Invitrogen, Carlsbad, CA). Growth media were changed at 24 h and supernatant containing lentivirus was harvested at 48 h. The harvested supernatant was further concentrated between 25× and 100×, by centrifugation (3000 rpm for 15 min at 4°C) using Centricon Plus-20 Centrifugal Filter Units (Millipore, Billerica, MA) and stored at -80°C before use. Raw264.7 cells were cultured in DMEM media supplemented with 10% fetal bovine serum and 1% antibiotic-antimycotic (Invitrogen, Carlsbad, CA) at 37°C in a humidified atmosphere of 5% CO_2_. Raw264.7 cells were transduced with the retrovirus to express both effluc and Thy1.1 genes. Thy1.1-positive cells were sorted using CD90.1 microbeads (Miltenyi Biotec, Bergisch Gladbach, Germany).

Reverse transcription polymerase chain reaction (RT-PCR) analysis

Raw264.7 cells and Raw-luc cells were lysed using a Trizol reagent (Invitrogen, Carlsbad, CA), and the total RNA was extracted according to the manufacturer’s protocols. Reverse transcription was performed using the Revert-Aid First Strand cDNA Synthesis Kit (Fermentas, Ontario, Canada). In brief, 2 µg of total RNA were reverse-transcribed in a final volume of 20 μl containing 1 μl oligo(d)T primer, 4 μl 5× reaction buffer, 2 μl 10 mM dNTP mix, and 1 μl MuLV reverse transcriptase (Fermentas, Burlington, Ontario, Canada). The effluc gene was amplified with forward (5’-GCACAAGGCCATGAAGAGAT-3’) and reverse (5’-CTTCTTGCTCACGAACACCA-3’) primers and 2 units of Taq DNA polymerase (Takara, Shiga, Japan) using a GeneAmp PCR system (Bio-Rad, Hercules, CA,). After denaturation of the samples for 1 minute at 94°C, 30 cycles of 25 sec at 94°C, 30 sec at 57°C, and 30 sec at 72°C were followed with an additional 10 min at 72°C. Samples were separated by electrophoresis in an ethidium bromide-stained agarose gel.

In vitro Luciferase assay

To perform luciferase assays, 0.5 × 10^4^, 1 × 10^4^, 2 × 10^4^ and 4 × 10^4^ Raw cells and Raw-luc cells were seeded into 96-well plates and cultured with DMEM supplemented with 10% FBS. After 24 h of additional culture, 100 µg/ml D-luciferin (PerkinElmer, Santa Clara, CA) was added to each well, and the relative light unit was measured using a microplate luminometer (Molecular Devices, Sunnyvale, CA) for 1 second.

In Vitro cell proliferation assay

Rates of cell proliferation were determined using a Cell Counting Kit-8 (Dojindo Laboratories, Tokyo, Japan). Raw cells and Raw-luc cells were plated in 96-well plates (2× 10^4^ cells per well). Two days later, 10 µl of ‘Cell Count Solution’ was added to each well, and the plates were incubated at 37°C for 3 h. The absorbance was measured at 450 nm using a microplate reader (Bio-Rad Laboratories, Hercules, CA).

In vivo bioluminescence imaging of subcutaneously implanted Raw-luc cells

For in vivo characterization studies, 2 × 10^5^, 1 × 10^6^ and 5 × 10^6^ Raw-luc cells were subcutaneously inoculated into the right upper, left lower and right lower flank of nude mice (n = 3). D-luciferin (150 mg/kg) dissolved in PBS (pH 7.5) was administered intraperitoneally at 30 min after the cell inoculation. At 10 minutes after D-luciferin injection, bioluminescence imaging (600 seconds acquisition) was performed using the IVIS Lumina II imaging system (PerkinElmer, Waltham, MA).

**References**

[1] Rabinovich BA, Ye Y, Etto T, Chen JQ, Levitsky HI, et al. (2008) Visualizing fewer than 10 mouse T cells with an enhanced firefly luciferase in immunocompetent mouse models of cancer. Proc Natl Acad Sci USA 105: 14342–6.
