# Supplementary material for: Characterization of Partial Ligation-Induced Carotid Atherosclerosis Model Using Dual-Modality Molecular Imaging in ApoE Knock-out Mice
Source: PLoS One. 2013 Sep 12;8(9):e73451. doi: 10.1371/journal.pone.0073451 (PMC3772018; doi:10.1371/journal.pone.0073451)
Supplement: File S2 — (PDF) [file pone.0073451.s002.pdf]

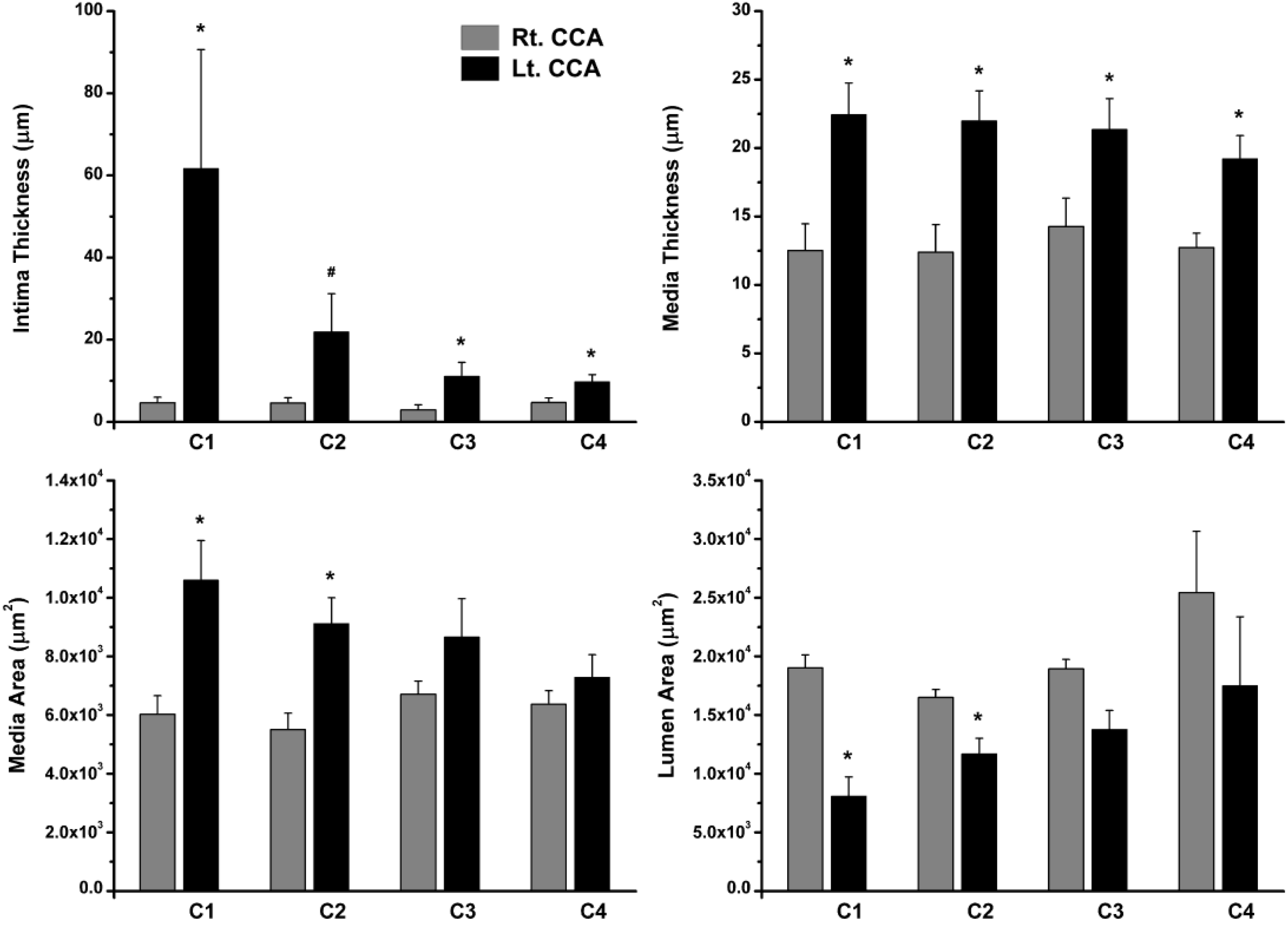

Rt. CCA
Lt. CCA
Intima Thickness (μm)
Media Thickness (μm)
Media Area (μm^2)
Lumen Area (μm^2)
C1
C2
C3
C4
*
#
0
20
40
60
80
100
5
10
15
20
25
30
0.0
2.0x10^3
4.0x10^3
6.0x10^3
8.0x10^3
1.0x10^4
1.2x10^4
1.4x10^4
5.0x10^3
1.5x10^4
2.0x10^4
2.5x10^4
3.0x10^4
3.5x10^4

Figure S1. Quantification of the degree of vascular remodeling two weeks after partial ligation of the left common carotid artery (CCA) in $ApoE^{-/-}$ mice on a high fat diet. When it goes down from the ligated left distal CCA segment (C1) to the proximal CCA origin (C4), there is a trend towards decreasing (the longest) intima or media thickness and increasing lumen area. In the left CCA, compared with the contralateral right CCA, intima or media thickness is higher, media area is larger, and lumen area is smaller ($^{*}p < 0.05$ and $^{\#}p < 0.1$, Wilcoxon signed-rank test).

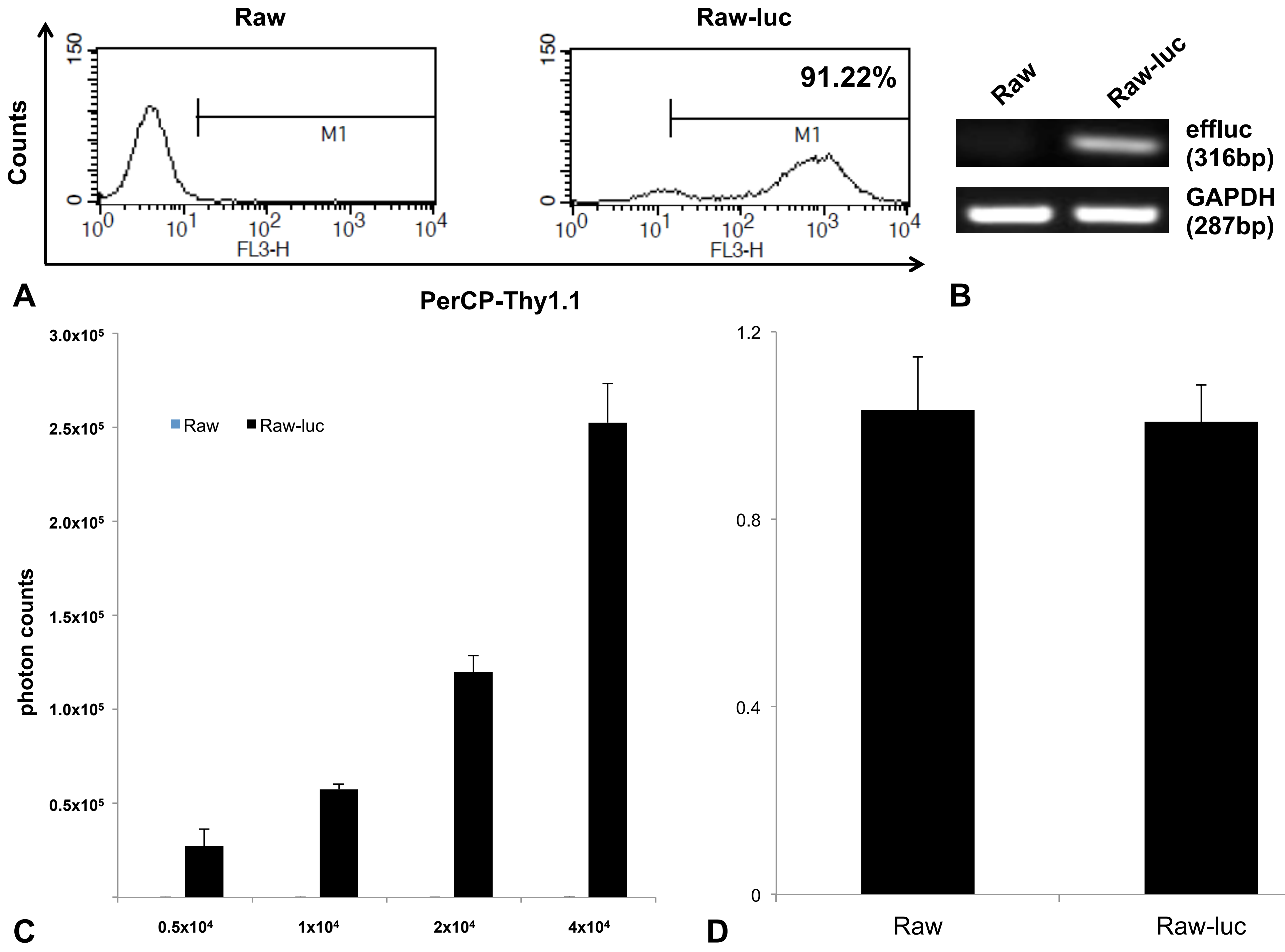

Raw
Raw-luc
Counts
150
0
M1
91.22%
FL3-H
PerCP-Thy1.1
effluc (316bp)
GAPDH (287bp)
A
B
photon counts
3.0x10⁵
2.5x10⁵
2.0x10⁵
1.5x10⁵
1.0x10⁵
0.5x10⁵
0.5x10⁴
1x10⁴
2x10⁴
4x10⁴
1.2
0.8
0.4
0
C
D

Figure S2. Stable expression of enhanced firefly luciferase (effluc) gene in Raw264.7 cells. Raw-luc cell line was established by transducing with the retrovirus to express both effluc and Thy1.1 genes. Fluorescence-activated cell sorting analysis shows that Raw-luc cells highly express Thy1.1 gene (A). RT-PCR analysis of Raw-luc cells reveals a fragment with a length of 316 bp, reflecting the incorporation of effluc gene; the band was not detected from parental Raw cells (B). The luciferase activity of Raw-luc cells was about 4000-fold higher compared with that of untransfected Raw cells ($2.5\times10^5 \pm 8.7\times10^4$ vs. 58.0 ± 9.8 relative luminescence units / mg protein, $p < 0.01$) (C). There was no significant difference in cellular proliferation between Raw cells and Raw-luc cells (D).

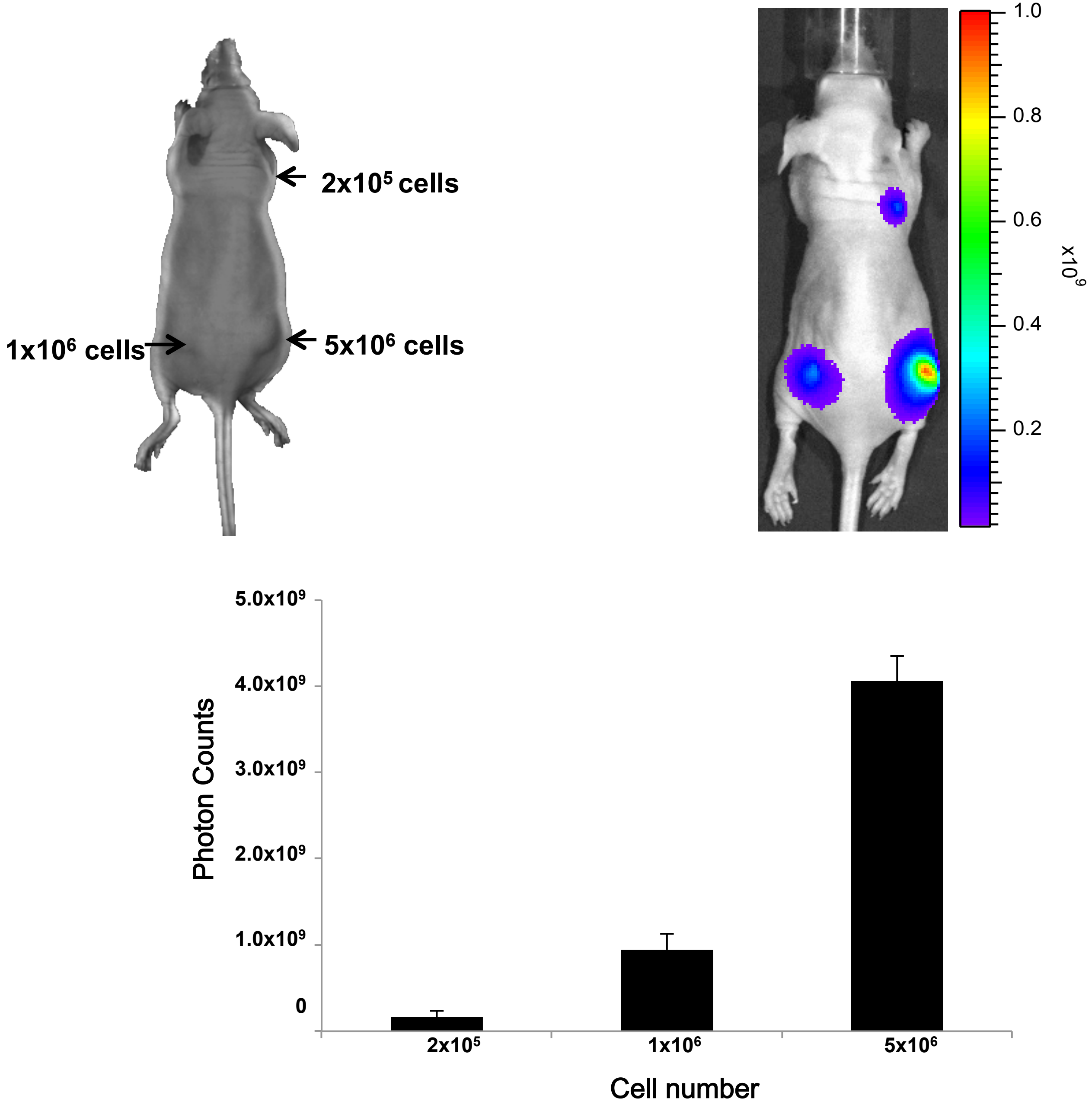
2x10^5 cells
1x10^6 cells
5x10^6 cells
1.0
0.8
0.6
0.4
0.2
x10^9
5.0x10^9
4.0x10^9
3.0x10^9
2.0x10^9
1.0x10^9
0
Photon Counts
2x10^5
1x10^6
5x10^6
Cell number

Figure 3S. In vivo bioluminescence imaging of subcutaneously injected Raw-luc cells, with pseudo-color overlay (photon counts / second). To determine whether Raw-luc cells could be visualized in a living organism, bioluminescence imaging was performed at 30 minutes after subcutaneous implantation of different numbers of Raw-luc cells. Strong bioluminescent signal is observed at each inoculation site, and the signal intensities correlate with the numbers of Raw-luc cells.

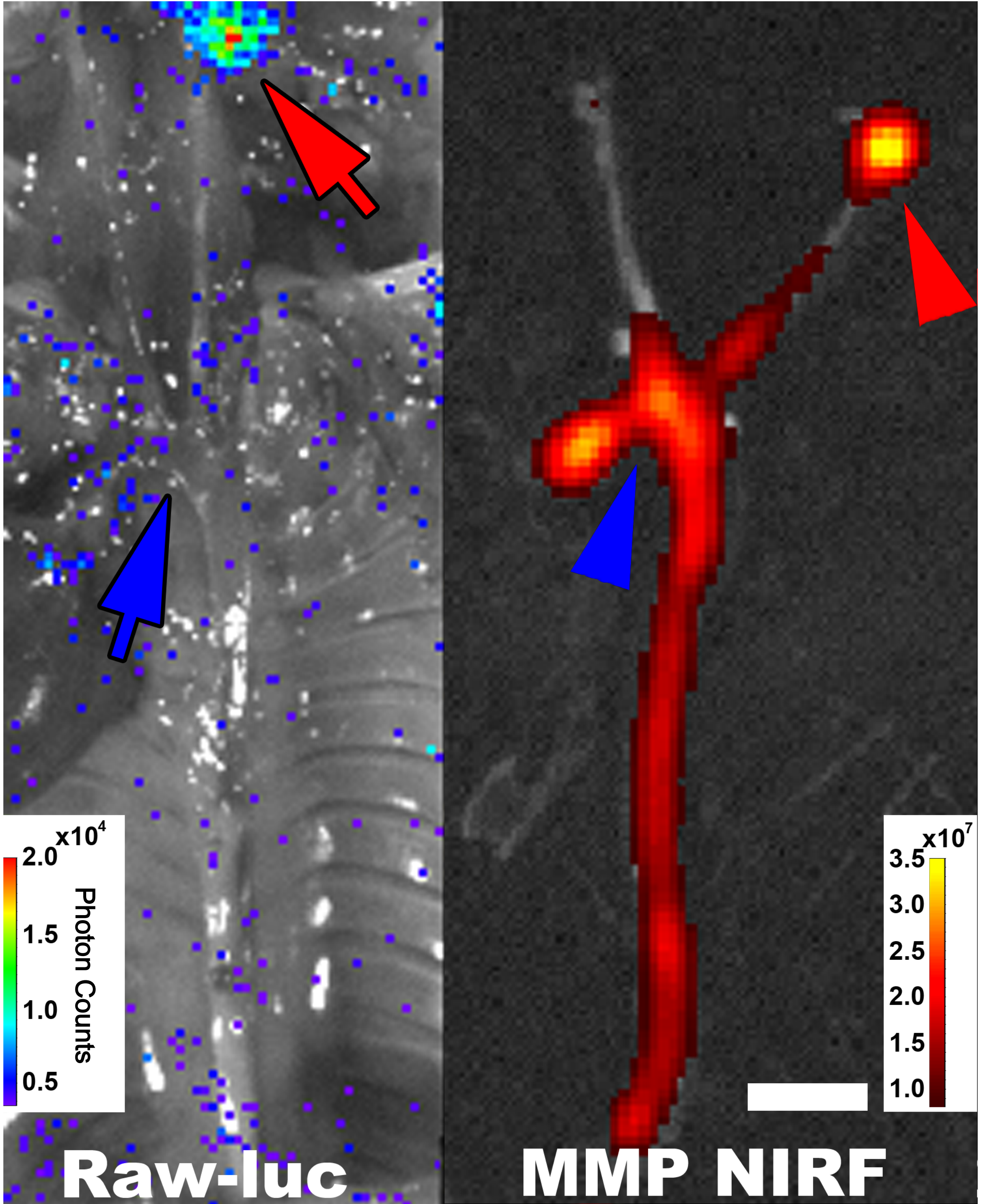

x10^4
2.0
1.5
1.0
0.5
Photon Counts
Raw-luc
x10^7
3.5
3.0
2.5
2.0
1.5
1.0
MMP NIRF

Figure 4S. Ex vivo dual-modality molecular imaging (with pseudo-color overlay) using an activatable matrix metalloproteinase (MMP)-2/9 probe and Raw-luc macrophages. Two weeks after partial ligation of the left common carotid artery in $ApoE^{-/-}$ mice on a high fat diet, intravenously-injected Raw-luc macrophages were recruited to the ligated CCA area (red arrow) rather than to the aortic arch (blue arrow). However, strong MMP-2/9-related near-infrared fluorescent (NIRF) signal (arbitrary unit) is observed in both areas (red and blue arrow-heads). Scale-bar, 1mm.
